# Systematic review of utility values used in the pharmacoeconomic evaluations for schizophrenia: implications on cost-effectiveness results

**DOI:** 10.1080/20016689.2019.1648973

**Published:** 2019-08-22

**Authors:** Junwen Zhou, Aurélie Millier, Clément François, Samuel Aballéa, Mondher Toumi

**Affiliations:** a Public Health Department, Aix-Marseille University, Marseille, France; b Health Economic and Outcome Research Department, Creativ-Ceutical, Paris, France; c Health Economic and Outcome Research Department, Creativ-Ceutical, Rotterdam, Netherlands

**Keywords:** Utility, schizophrenia, antipsychotics, pharmacoeconomics, economic model

## Abstract

**Background and Objectives**: Utility elicitation studies for schizophrenia generate different utility values for the same health states. We reviewed utility values used in schizophrenia pharmacoeconomic evaluations and evaluated the impact of their selection on the incremental cost-effectiveness ratio (ICER).

**Methods**: A systematic search was performed in Medline and Embase. Health state definitions, associated utility values, elicitation studies, and value selection processes were extracted. Sets of utility values for all schizophrenia health states were used in a cost-effectiveness model to evaluate the ICER.

**Results**: Thirty-five cost-utility analyses (CUAs) referring to 11 utility elicitation studies were included. The most frequent health states were ‘stable’ (28 CUAs, 7 utility elicitation studies, 10 values, value range 0.650–0.919), ‘relapse requiring hospitalisation’ (18, 5, 7, 0.270–0.604), ‘relapse not requiring hospitalisation’ (18, 5, 10, 0.460–0.762), and ‘relapse only’ (10, 5, 6, 0.498–0.700). Seventeen sets of utility values were identified with difference in utility values between relapse and stable ranging from −0.358 to −0.050, resulting in ICERs ranging from −56.2% to +222.6% from average.

**Conclusion**: The use of utility values for schizophrenia health states differs among CUAs and impacts on the ICER. More rigorous and transparent use of utility values and sensitivity analysis with different sets of utility values are suggested for future CUAs.

## Introduction

Health state utility data are widely used in pharmacoeconomic evaluation. By weighting the lifetime spent in a specific health state over time with the utility value associated with that health state, researchers could measure the impact of pharmacotherapy in terms of quality-adjusted life year (QALY), which is considered an ideal measure of health effect for benefit assessment in an economic evaluation [1]. Cost-utility analysis (CUA), a type of pharmacoeconomic evaluation which employs QALY as a health outcome, is preferred in many countries which require health technology assessment (HTA) for the drug reimbursement, such as the UK [2], France [3], Canada [4], and Japan [5].

CUA as part of pharmacoeconomic evaluation may play a role in obtaining reimbursement for treatments of schizophrenia. This type of analysis takes into consideration all the driver of QALY such as treatment efficacy and treatment side effects. In the case of schizophrena, it could include the treatment benefits such as stabilisation of the disease and prevention of relapses, the treatment side effects such as extrapyramidal symptoms (EPS) and metabolic symptoms, and the other QALY drivers. There has been a growing number of pharmacoeconomic evaluations performed with CUA in schizophrenia. A previous review [6] identified that 48 out of 79 model-based pharmacoeconomic evaluations in schizophrenia included CUAs. The proportion of CUAs was more than two-fold in 2008–2016 than that in 2000–2007 (79% vs. 33%) [6].

Although many CUA were performed for pharmacoeconomic evaluations in schizophrenia, different utility values associated with the same health state were used to estimate the QALY gained [6]. The heterogeneity in using utility values could be due to the different selection of utility elicitation studies which had different study designs, such as elicitation methods and responders. The utility values could be very different if they were generated from different elicitation methods. Noel et al found the inconsistency between anyone of the direct elicitation methods by standard gambling and time trade-off, and anyone of visual analogue scale and the indirect elicitation methods by EQ-5D and health utility index [7]. Briggs et al also found that time-trade off elicited a inconcordant utility weights from EQ-5D [8]. The heterogeneity could also come from selecting different values reported within the utility elicitation studies where the utility values were based on different types of responders (layperson, patient, psychiatrist, etc.) [9,10] and calculated using different utility elicitation methods (time-trade off and regression) [9].

Reviews from the literature related to utility values for schizophrenia focused only on the methodologies and results from the utility elicitation studies. The National Institute for Health and Clinical Excellence (NICE) [11] reviewed and selected the utility values to be used in their models. Mavranezoli *et al* [12]. identified and criticised the utility elicitation studies for schizophrenia-related health states in terms of methodologies and results. Furthermore, Nemeth *et al* [13]. identified and described three utility elicitation studies using different approaches to convert Positive and Negative Syndrome Scale (PANSS) scores [14] into health states utilities. However, how utility values for the health states of schizophrenia were used in the pharmacoeconomic evaluations remains unclear. In addition, the different selection of utility values for the health states could be influential on the cost-effectiveness results, which could be true in the other disease areas as well. Therefore, this study was conducted to identify the pattern of utility values for the health states of schizophrenia used in the CUAs for pharmacoeconomic evaluations in schizophrenia and its impact on the cost-effectiveness results.

## Methods

A systematic search was performed to identify pharmacoeconomic evaluation models in schizophrenia reporting the utility values. A previous systematic review [6] of pharmacoeconomic evaluation models in schizophrenia was used to identify CUAs reporting utility values for the health states of schizophrenia. The search results of the previous search in April 2016 (from January 2000 to April 2016) were updated in April 2019 (from April 2016 to April 2019). The same search strategy in the previous review was used by searching terms related to schizophrenia, pharmacoeconomic evaluation and model in MEDLINE and EMBASE. The search results were screened to identify the schizophrenia pharmacoeconomic evaluation models. The identified models were pooled with the models identified in the previous search for a further screening to identify the CUAs reporting the utility values for states of schizophrenia. Trial-based CUAs were not considered since they rather collect utilities than elicit utilities during the trials, whereas model-based CUAs commonly use elicited utility weights to synthesise the multi-dimension impact of treatment [15]. Studies using disability weight were excluded, as disability weight is a different quality of life assessment framework from utility weight [16].

The following information was extracted from each included study: health states and their detailed definitions (when available), utility values for each health state, and associated sources. The associated source of utility values was utility elicitation study. If a CUA was referred as source of utility values, the references of this CUA would be screened to identify the utility elicitation study which generated these utility values. Also, the approach of utility values selection from the source was extracted, including the target country/region, utility elicitation methods, the responder, and the interpretation of the health states from the utility elicitation study into the health states used in the CUA. If the selection approach was not reported, the potential approach would be extracted by matching the utility values used in the CUA with the utility values reported in the utility elicitation study.

The following information was summarised: number of CUAs, number of utility values per health state, number of source utility elicitation studies, and the utility values for each health state of schizophrenia. A most commonly used utility value would be presented rather than the mean/median utility value, since the utility value reported in CUAs was the result of a discrete selection process from multiple sources. Utility values for all the health states of schizophrenia within each CUA were considered as one set of utility values. The set of utility values was summarised with the source utility elicitation studies, country/region, the approach used, responder, and possible health state interpretation.

**Figure 1.**
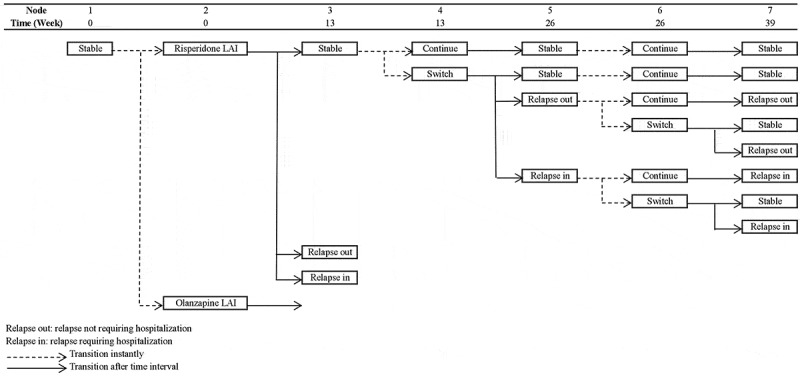
Model structure.

To evaluate the impact of the selection process of utility values for the health states of schizophrenia, the model structure developed by Einarson *et al* [17]. was replicated and used to test the result of our review. This model was a decision tree comparing the 1-year economic consequence of 3-line antipsychotic treatment sequences. It was selected according to the following reasons: 1) its structure was widely used with eight adaptations [17–24]; 2) it did not consider utility values for the health states other than schizophrenia, such as treatment-related side effects; 3) it was a simple model requiring fewer interpretations to replicate the model. Our analysis only compared olanzapine long-acting injection (LAI) with risperidone LAI as a first-line treatment, since this comparison generated non-dominance ICER results in Einarson *et al* [17]. The model was re-constructed based on the reported structural information (Figure 1). The transition probability data and the cost results were obtained from Einarson *et al* [17]. The utility values for the health states of stable, relapse requiring hospitalisation, and not requiring hospitalisation were selected from the identified sets of utility values for the health states of schizophrenia.

## Results

**Figure 2.**
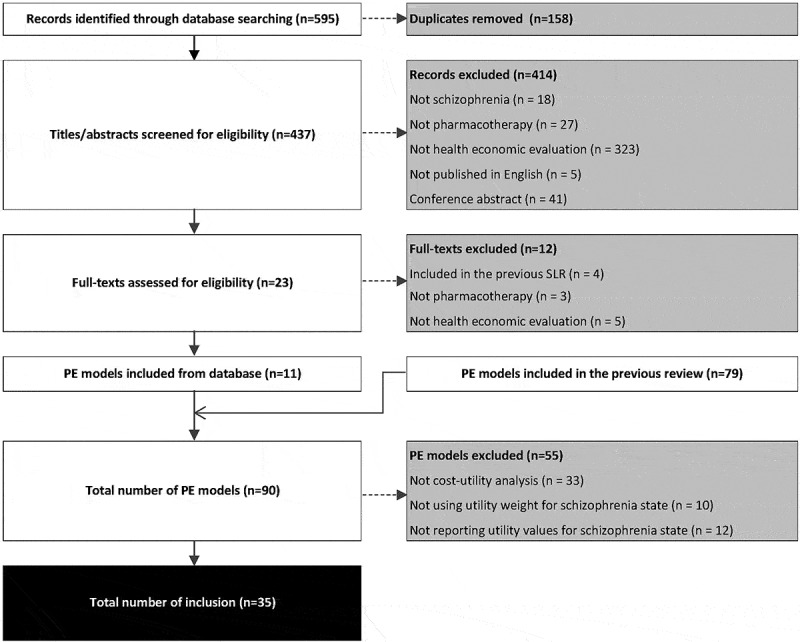
PRISMA diagram of the search. PE: pharmacoeconomic evaluation.

After screening 595 publications, the present review identified an additional 11 pharmacoeconomic evaluation models which were not included in the 79 models identified in the previous review [6]. Out of 90 studies, 35 reported detailed utility values for the health states of schizophrenia and were included in the present analysis [17–50] (Figure 2). The other 55 were excluded as they were not CUA, not using a utility weight for the health state of schizophrenia, or not reporting detailed utility values. The characteristics of the newly identified CUAs [23,24,48–50] are presented in Supplement 1; the others can be found in the previous review [6]. The total CUAs included were conducted in 16 countries/regions (mostly in the USA, n = 8), with cohort-level models (16 Markov models and 15 decision tree models). They all compared pharmacotherapy as first-line treatment for general schizophrenia (n = 33) or treatment resistant schizophrenia (n = 2), in terms of incremental cost per QALY gained from the payer’s perspective. The timeframes were mostly 1-year (n = 20), followed by 5-year (n = 7), lifetime (n = 5), and 10-year (n = 4).

### Utility values for the health states of schizophrenia

The CUAs (n = 28) mainly classified the health states of schizophrenia into stable and relapse, with some CUA further classifying the health states of relapse into relapse requiring hospitalisation and relapse not requiring hospitalisation. Other CUAs either classified the health states of schizophrenia into mild symptom and moderate symptom, or directly applied overall schizophrenia as a health state to assess the impact of the side effect by antipsychotics. Overall there were ten health states of schizophrenia with utility values reported by CUAs. The health state of stable (n = 28), relapse requiring hospitalisation (n = 18), relapse not requiring hospitalisation (n = 18), and relapse (n = 10) were mostly used (Table 1). The most numbers of utility values was reported in the health state of stable (n = 10; range from 0.650 to 0.919), followed by relapse not requiring hospitalisation (10; 0.460–0.762), relapse requiring hospitalisation (7; 0.270–0.604), relapse (5; 0.498–0.700). The other six health states had the number of values ≤4 and a difference of value range ≤0.5. The combination of hospitalisation status (Yes/No) with the health states of schizophrenia was considered as a health state of schizophrenia since it was primarily used to represent the disease severity. There were eight CUAs reporting the utility values for the health states of schizophrenia in combination with three other types of state: antipsychotic treatment (n = 4), adherence level (n = 3), and state duration (n = 1).

**Table 1.** Utility values for the health states of schizophrenia in the CUAs.

	Number of				
Schizophrenia health state	CUAs considering the health state	Utility values used to document the health state	Sources	Most commonly used utility value	Range of utility values
Stable^*	28	10	7	0.890	0.650–0.919
Relapse outpatient^*	18	10	5	0.659	0.460–0.762
Relapse inpatient^	18	7	5	0.490	0.270–0.604
Relapse^&^	10	5	4	0.670	0.498–0.700
Overall schizophrenia	3	2	3	0.730	0.730–0.750
Mild*	2	4	2	0.910	0.860–0.910
Mild outpatient*	2	2	1	0.860, 0.910	0.860–0.910
Mild inpatient*	2	2	1	0.840, 0.870	0.840–0.870
Moderate	2	2	2	0.820, 0.830	0.820–0.830
Moderate inpatient	2	1	2	0.820	0.820

^: 3 CUAs applied 2–3 values in combination with adherence level (full, partial, none); ^&^: 1 CUA applied 3 values in combination with state duration (<6 months, 6–12 months, >12 months),; *: 3 CUAs applied 2–3 values in combination with antipsychotic treatments (typicals, atypicals other than clozapine, clozapine); CUA: cost-utility analysis; relapse/mild outpatient: relapse/mild not requiring hospitalization; relapse/mild/moderate inpatient: relapse/mild/moderate requiring hospitalization

### Selection of source of utility value

Most CUAs (n = 31) just reported the source of the used utility values without specifying the process of selecting the source. Of them, most derived the utility values from published literatures, and 2 through interviews [42,43], and 1 through database analysis [29]. The other four studies [11,26,35,50] reported a systematic review process before the selection of the source, with 2 [26,35] identifying the utility elicitation studies through reviewing economic evaluations, and another 2 [11,50] through reviewing utility elicitation studies.

In total, eleven utility elicitation studies [9,10,29,42,43,51–56] were used. Lenert *et al* [54]. was mostly referred (n = 18), whether for stable (n = 17), relapse not requiring hospitalisation (n = 13) and relapse (n = 4) health states. Revicki *et al* [10]. was mostly referred for the health state of relapse requiring hospitalisation (n = 9). Utility values for mild and moderate symptoms health states were derived from two studies by Oh *et al*. [42,43] and one study by Glennie et *al* [52].; each applied in ≤2 CUAs. Utility values for the health state of overall schizophrenia were derived from Konig *et al* [53]., Lenert *et al* [54]. and Seong *et al* [56].; each applied in 1 CUA. Most of the CUAs (n = 27) derived the utility values for all the health states of schizophrenia from a single utility elicitation study, referring ten utility elicitation studies in total, and the others derived them from a same group of studies composed by five utility elicitation studies.

### Selection of utility value from source

The CUAs using multiple sources applied a simple average of the utility values from the sources, and reported just the pooled values and the sources. Most of the CUAs using single source just reported the utility value and the source, with only two [11,27] studies reporting further information on the interpretation of the health states from the utility elicitation studies into the health states used in the CUA.

Five of the source utility elicitation studies have multiple utility values for the same health states due to either the difference in the target country/region, elicitation method, and responder. Only the CUAs referring Briggs et al were found to derive different utility values due to the different selection of the elicitation methods. (Table 2)

**Table 2.** Characteristics of the utility elicitation studies referred in the CUAs.

Study	Country/region	Elicitation method	Responder	Schizophrenia health state
Briggs 2008[9]	UK	TTO*	patient*	stable, relapse
			layperson	
		regression parsimonious*	NR	
		regression unrestricted	NR	
Cummins 1998[51]	UK	IHQL	NR	response, response then relapse, no response
Dilla 2014[29]	Europe	regression	NR	no relapse, relapse
Glennie 1997[52]	Canada	SG	patient	mild, moderate
König 2009[53]	Germany*	EQ-5D	patient	schizophrenia
	UK			
Lenert 2004[54]	US	SG weighted*	general	state 1–8 (or mild, moderate 1–2, severe 1–4, extremely severe)
		SG unweighted		
Oh 2001a[42]	Canada	SG	patient	mild-outpatient, mild-inpatient, moderate-inpatient
Oh 2001b[43]	Canada	SG	patient	mild, moderate
Osborne 2012[55]	Australia	TTO	general	relapsed/untreated, well-controlled/treated
Revicki 1996[10]	UK	SG*	physician*	outpatient (excellent, good, moderate, negative), inpatient (acute positive), current (remission)
		PC	patient	
			caregiver	
Seong 2004[56]	Korean	EQ-5D	general	other disease

*: the country/region/elicitation methods/responder that generated the utiltiy value used in the cost-utility analyses when there are multiple options

UK: UK; US: USA; TTO: time trade-off; IHQL: index of health related quality of life; SG: standard gamblingl; EQ5D: EuroQol 5 dimensions; PC: paired comparison; NR: not reported

Only eight CUAs used the same health states as the health states defined in the utility elicitation studies. Of them, five CUAs derived the utility values from the other sources, with two applying stable and relapse health states referring Brigg et al [9], two applying mild and moderate symptom health states referring Oh et al [42] and Glennie et al [52], and one applying the health state of overall schizophrenia referring Konig et al [53]. In total, the CUAs used twenty sets of utility values for all the health states of schizophrenia. (Table 3)

**Table 3.** The interpretation of health states in utility elicitation studies into the health states in CUAs.

Utility study	CUA health state	Value set	Schizophrenia health state in the utility elicitation study			No. CUA
CUA schizophrenia health state: stable, relapse			stable (schizophrenia)	relapse (outpatient)	relapse inpatient	
Lenert[54]	Stable, relapse outpatient/inpatient	# 1	state 1 or 2*	state 3 or 4*	state 6 or 8*	2[25,31]
		# 2	state 1 or 2*	state 3 or 5*	state 6 or 8*	1[32]
		# 3	state 1	state 3	state 6	1[48]
		# 4	state 1	state 2–3 (average)	state 4–8 (average)	1[34]
	Stable, relapse	# 5	state 1–3 (40%, 30%, 30%)	state 7–8 (60%, 40%)	-	4[11,33,39,50]
	Overall schizophrenia	# 6	state 3	-	-	1[38]
Revicki[10]	Stable, relapse outpatient/inpatient	# 1	outpatient (excellent)	outpatient (negative)	inpatient (acute positive)	1[27]
	Stable, relapse	# 2	outpatient (excellent)	inpatient (acute positive)	-	2[45,46]
		# 3	current (remission)	inpatient (acute positive)	-	1[40]
Briggs (TTO)[9]	Stable, relapse outpatient/inpatient	# 1	stable	stable, relapse (average)	relapse	3[30,41,47]
Briggs (Regression)[9]	Stable, relapse	# 2	stable	relapse	-	2[28,44]
Osborne[55]	Stable, relapse outpatient/inpatient	# 1	well-controlled/treated	treated, untreated (average)	relapse/untreated	1[23]
Dilla[29]	Stable, relapse	# 1	no relapse	relapse	-	1[29]
König[53]	Overall schizophrenia	# 1	schizophrenia spectrum disorders	-	-	1[36]
Seong[56]	Overall schizophrenia	# 1	other disease	-	-	1[37]
Multiple[9,10,43,51,54]	Stable, relapse outpatient/inpatient	# 1	Average of 5 references[9,10,43,51,54]	Average of 2 references[10,54]	Average of 2 references[10,43]	7[17–22,50]
		# 2	Average of 5 references[9,10,43,51,54]	stable, relapse (average)	Average of 2 references[10,43]	1[24]
CUA schizophrenia state: mild, moderate symptom			mild (outpatient/inpatient)	moderate (inpatient)		
Oh a[42]	mild, moderate outpatient/inpatient	# 1	mild–inpatient, mild–outpatient	moderate–inpatient	-	2[35,42]
Glennie[52]	mild, moderate	# 1	mild	moderate	-	1[26]
Oh b[43]	mild, moderate	# 1	mild	moderate	-	1[43]

*: the interpretation of state depends on the adherence level (none, partial, full); relapse/outpatient: relapse not requiring hospitalisation; relapse inpatient: relapse requiring hospitalisation; CUA: cost-utility analysis; TTO: time trade-off.

### Impact of the utility value selection process

As shown in Table 3, there were nine utility value sets covering the same three health states of schizophrenia as in Einarson *et al* [17].: stable, relapse requiring hospitalisation, and relapse not requiring hospitalisation. There were another eight utility value sets that could differentiate the health states of schizophrenia: five for stable and relapse health states and three for mild and moderate symptoms health states. To fit them in the model, the following modifications were made: 1) the health states of mild and moderate symptoms were assumed to be the same health state as stable and relapse respectively; 2) the health states of relapse requiring hospitalisation and relapse not requiring hospitalisation were assumed to have the same utility value as the health state of relapse when the utility value was available only in the health state of relapse. When multiple utility values were reported for the health states of schizophrenia in combination with the other types of states, an average value was used.

After the modification, seventeen utility value sets for the health states of schizophrenia were generated, with the difference in utility value between the health states of relapse and stable ranging from −0.358 to −0.050, and the utility value for the health state of stable ranging from 0.659 to 0.919. The application of the utility value sets in the model resulted in an incremental QALY ranging from 0.016 to 0.002 and an ICER ranging from 62,302 to 459,293 euro per QALY gained (Table 4). The average incremental QALY and average ICER were 0.010 and 142,384 Euro per QALY gained respectively. ICER moved the Swedish willingness to pay threshold (55,000 Euro per QALY gained [57]) from 1.13 times to 8.35 times. The ranges of percentage changed from the average results: 38.2% and 36.2% for the total QALY in reference and comparator, 132.4% for the incremental QALY and 278.8% for the ICER.

**Table 4.** Impact of different utility value sets for the health states of schizophrenia.

	Utility value		QALY		QALY	ICER (Euro)
Utility value set	Relapse (outpatient, inpatient) vs. Stable	Stable	Risperidone LAI	Olanzapine LAI	Olanzapine LAI vs. Risperidone LAI	
**Individual results**						
Briggs # 2	−0.358*	0.856	0.765	0.780	0.015	64,147
Multiple # 1	−0.316 (−0.231, −0.400)	0.890	0.801	0.817	0.016^#^	62,302^#^
Osborne # 1	−0.304 (−0.203, −0.405)	0.675	0.588^&^	0.603^&^	0.016	62,510
Multiple # 2	−0.300 (−0.200, −0.400)	0.890	0.804	0.819	0.015	63,291
Revicki # 2	−0.270*	0.830	0.762	0.773	0.011	85,054
Revicki # 1	−0.250 (−0.230, −0.270)	0.830	0.765	0.776	0.011	87,462
Lenert # 3	−0.245 (−0.140, −0.350)	0.880	0.807	0.820	0.013	73,845
Briggs # 1	−0.236 (−0.157, −0.315)	0.919	0.851	0.863	0.012	80,396
Lenert # 2	−0.235 (−0.130, −0.340)	0.815	0.745	0.758	0.013	76,299
Lenert # 1	−0.230 (−0.120, −0.340)	0.815	0.746	0.758	0.013	76,773
Lenert # 4	−0.223 (−0.135, −0.310)	0.880	0.815	0.826	0.012	82,760
Revicki # 3	−0.190*	0.750	0.702	0.710	0.008	120,867
Lenert # 5	−0.129*	0.799	0.766	0.772	0.005	178,020
Dilla # 1	−0.117*	0.770	0.740	0.745	0.005	196,840
Oh b # 1	−0.075*	0.905	0.886^#^	0.889^#^	0.003	306,195
Glennie # 1	−0.067*	0.887	0.870	0.873	0.003	344,470
Oh a # 1	−0.050*	0.870	0.857	0.859	0.002^&^	459,293^&^
**Summary results**						
Average			0.781	0.791	0.010	142,384
Best case (vs. average)			0.886 (+13.5%)	0.889 (+12.4%)	0.016 (+53.2%)	62,302 (−56.2%)
Worst case (vs. average)			0.588 (−24.7%)	0.603 (−23.7%)	0.002 (−79.2%)	121,554 (+222.6%)

*: Same utility value was used for both relapse requiring hospitalisation or not requiring hospitalisation; #: best case; &: worst case; Relapse outpatient: relapse not requiring hospitalisation; Relapse inpatient: relapse requiring hospitalisation;

ICER: incremental cost-effectiveness ratio; LAI: long-acting injection; QALY: quality-adjusted life year.

## Discussion

### Health states of schizophrenia and the associated utility values

The CUAs for pharmacoeconomic evaluation of schizophrenia were found to apply various utility values for the health states of schizophrenia. In the CUAs, the health state of schizophrenia were mainly classified into 1) stable and relapse or 2) stable, relapse requiring hospitalisation and relapse not requiring hospitalisation, with the greatest number of different utility values used in these health states. Some of the health states had utility values reported in a combination of the other type of states including adherence level, the antipsychotic treatment used, and state duration. Utility value for the health state of overall schizophrenia was considered in the studies which focused mainly on side effects of antipsychotic treatments [36,38]. Besides the difference in utility values for a single schizophrenia health state, the difference in utility value between the health states of stable and relapse also varies largely among the CUAs (−0.358 to −0.050), even if the same source of utility elicitation study was used (Brigg et al [9]: −0.358 to −0.236; Lenert et al [54]: −0.245 to −0.129; Revicki et al [10]: −0.270 to −0.190).

### Source of heterogeneity

The heterogeneity in utility values for each health state of schizophrenia used in CUAs was mainly rooted in selecting different utility elicitation studies. The utility elicitation studies differed in countries/regions, elicitation methods, responders and classifications of health state of schizophrenia, and consequently generated different utility values. This kind of heterogeneity could be normal, as there are multiple criteria to select the utility elicitation study for CUA. However, the heterogeneity could not be further explored, as almost all the CUAs did not report the other possible options, and did not justify their selection. Another source of heterogeneity was the different interpretation of the health states from the utility elicitation studies into the health states used in the CUAs. The CUAs referred the utility elicitation study by Lenert et al [54]. mostly, but none them developed their model structure with the same health states of schizophrenia as the health states defined in the study by Lenert [54], resulting in 6 types of interpretation of Lenert’s health states into their health states.

### Consequences on ICER

Using the same model and the exact same assumptions for all parameters, but a different set of utilities, ICER varies considerably. The range of difference was 37% in QALY, 132% in incremental QALY, and 279% in ICER. This level of difference in ICER could significantly influence ICER based reimbursement decisions. If the average ICER is the same as the common Swedish threshold value of €55,000 per QALY [57], the ICER could be €177,416 in the best case utility value set (multiple 1) and €24,066 in the worst case (Oh 1). If the €55,000 per QALY was generated from the best case, the ICER of the worst case could be as high as €405,460 per QALY. If there is no standard for the selection of utility value sets, there is a lot of room for modification on the utility values selection to generate the ICER results meeting the requirement by the HTA.

### Implication for the other diseases

The heterogeneity in utility values used in CUAs probably also exists in the other disease area. The current reviews on the utility values mainly focus on the utility elicitation studies rather than how utility elicitation studies are used in the CUA. However, as long as there is no standard to identify and apply the utility elicitation study to the CUA, and there are multiple utility elicitation studies available for that disease, the CUA would probably select different utility values and generate different ICERs. Utility value is one of the main model characteristics, and should be considered as as important as the other factors that can influence the ICER such as model structure, time horizon, efficacy data, etc [58,59].

### Limitation

This study has several limitations. First, the interpretation of schizophrenia health states in the utility elicitation studies into the health states defined in the CUAs were mainly assumed by matching the utility value used in the CUA with the utility value reported in the utility elicitation studies, as only 2 CUAs reported the interpretation process. However, the impact is only restricted on the heterogeneity in the interpretation, and our study concerns more about the results of interpretation (the used utility values) than the pattern of interpretation. Next, the re-constructed model was slightly different from the original model as the structural information was not completely reported in the original model. However, the aim of our model was to explore the impact of utility vlaue selection rather than to generate the precise estimates of the economic results. The minor difference between the re-constructed model and the original model would not influence our results. Last, modification were made when the health states of schizophrenia of the utility value sets did not completely match the health states used in the re-constructed model. However, the impact should be minor as the modification captured the major difference in utility value between schizophrenia health states, which is the main driver of the economic results.

### Recommendations for future studies

#### Utility elicitation study selection

Given the identified results, a rigorous and transparent selection process of utility elicitation studies should be applied in the CUA in schizophrenia or even in the other diseases. We suggest a systematic review to identify the relevant utility elicitation study to avoid the selection bias, and a predefined strategy to handle studies when there are multiple studies meeting the inclusion criteria. Depending on the objective of the analysis, the strategy could be keeping all the studies for pooling or for sensitivity analysis, or using subsequent criteria to keep only one study, as long as a reasonable justification is given. When an appropriate selection process is performed by other researchers, it is possible to use their selection results, if the researcher could justify that these results are the same utility elicitation studies as they need in the CUA. However, the results should be at least updated with the same process if the selection results were generated several years before. For example, three CUAs [33,39,50] selected utility values from Lenert *et al* [54]. following the selection process as the NICE study [11]. However, if they have updated the NICE selection process, they might tend to select the utility values from Briggs *et al* [9]., which were based on the UK patients. This study was not considered in the NICE model because it was published in 2008, after the finalisation of the NICE model.

#### Utility value selection

The detailed selection process of utility values from the utility elicitation study should be reported in the future CUAs. Currently, the CUAs listed only the utility elicitation study to indicate the source of utility values. However, some of the utility elicitation studies generated multiple results with different ways of analysis and the different responders. Also, interpretations of utility values from the utility reference were common in CUAs for schizophrenia. These examples show that it is not enough to just list the utility elicitation study. We suggest the CUA to report the country/region, elicitation method and responder that generated the used utility values even if there is only one option for each of them in the utility elicitation study, and to report the interpretation process if they have different health states than the health states defined in the utility elicitation study. The appropriate use of utility values from the utility elicitation studies has also been discussed by the other researchers, which can serve as a guide for the future CUAs [60,61].

#### Sensitivity analysis with utility value sets

We also suggest to perform scenario analyses with different utility value sets. It is common to perform sensitivity analyses around a single utility value to identify the impact of the parameter uncertainty. However, this only identifies the impact of the uncertainty around this parameter, estimated with a specific approach. When more than one relevant utility value sets are identified are appropriate for the CUA, it is important to consider them all in the scenario analyses.

#### Generation of new utility elicitation studies

Generation of new utility elicitation studies for the health states of schizophrenia is suggested. They should target on the health states of stable and relapse (requiring hospitalisation or not) since they are the health states commonly used in the CUAs. Among the utility elicitation studies, only Briggs *et al* [9]. generated these utility values. There is also a need to generate utility values within the region/country where the CUA targets since the utility weight represents the preference of people and could be very different between countries/regions.

### Conclusion

The summary of utility values for the health states of schizophrenia used in CUAs for the pharmacoeconomic evaluation revealed large heterogeneity in the use of these utility values. The main sources of heterogeneity included a different selection of utility elicitation studies and a different interpretation of the health states defined in the source into the health states used in the CUA. Using different utility values have an important impact on the ICER possibly moving it from far below the accepted threshold to far above and making room for modifications in order to meet the HTA requirements on economic evidence. Consequently, a more rigorous and transparent process of selecting utility values and a sensitivity analysis of different utility values is suggested for the future CUAs.

## Supplementary Material

Supplemental Material
