# Supplementary material for: Systematic review of utility values used in the pharmacoeconomic evaluations for schizophrenia: implications on cost-effectiveness results
Source: J Mark Access Health Policy. 2019 Aug 22;7(1):1648973. doi: 10.1080/20016689.2019.1648973 (PMC6713214; doi:10.1080/20016689.2019.1648973)
Supplement: Supplemental Material [file ZJMA_A_1648973_SM0645.docx]

Supplement 1. Characteristics of newly included CUA studies

| Study | Model | Analysis | Country/Regions | Perspective | Population | Comparators | Outcome | Timeframe |
| --- | --- | --- | --- | --- | --- | --- | --- | --- |
| Aigbogun 2018^1^ | DT | CUA | United States | payer perspective | stable schizophrenia | brexpiprazole oral, cariprazine oral, lurasidone oral | cost per QALY gained | 1-year |
| Einarson 2017a^2^ | DT | CUA | Spain | payer perspective | stable schizophrenia | PP3M, PP1M | cost per QALY gained | 1-year |
| Einarson 2017b^3^ | DT | CUA | Netherland | insurer perspective | stable schizophrenia | PP3M, PP1M, haloperidol LAI, risperidone LAI, olanzapine oral | cost per QALY gained | 1-year |
| Nuhoho 2018^4^ | CLMM | CUA | United Arab Emirates | public payer perspective | stable schizophrenia | PP1M, oral antipsychotics | cost per QALY gained | 1-year |
| Rajagopalan 2016^5^ | CLMM | CUA | Scotland and Wales | healthcare services perspective | acute schizophrenia | lurasidone, aripiprazole | cost per QALY gained | 10-year |

CLMM: cohort-level Markov model; CUA: cost-utility analysis; DT: decision tree; LAI: long-acting injection; PP1M: paliperidone prescribed monthly; PP3M: paliperidone prescribed every 3 months; QALY: quality-adjusted life year;

References

1. Aigbogun MS, Liu S, Kamat SA, Sapin C, Duhig AM, Citrome L. Relapse prevention: a cost-effectiveness analysis of brexpiprazole treatment in adult patients with schizophrenia in the USA. ClinicoEcon 2018;10:443-56.

2. Einarson TR, Bereza BG, Garcia Llinares I, Gonzalez Martin Moro B, Tedouri F, Van Impe K. Cost-effectiveness of 3-month paliperidone treatment for chronic schizophrenia in Spain. Journal of Medical Economics 2017;20:1039-47.

3. Einarson TR, Bereza BG, Tedouri F, Van Impe K, Denee TR, Dries PJT. Cost-effectiveness of 3-month paliperidone therapy for chronic schizophrenia in the Netherlands. Journal of Medical Economics 2017;20:1187-99.

4. Nuhoho S, Saad A, Saumell G, Ribes D, El Khoury AC. Economic evaluation of paliperidone palmitate once monthly for treating chronic schizophrenia patients in the United Arab Emirates. Current Medical Research & Opinion 2018;34:601-11.

5. Rajagopalan K, Trueman D, Crowe L, Squirrell D, Loebel A. Cost-Utility Analysis of Lurasidone Versus Aripiprazole in Adults with Schizophrenia. Pharmacoeconomics 2016;34:709-21.
